# Erasing “bad memories”: reversing aberrant synaptic plasticity as therapy for neurological and psychiatric disorders

**DOI:** 10.1038/s41380-025-03013-0

**Published:** 2025-04-10

**Authors:** Zhuoyue Shi, Kailong Wen, Nabilah H. Sammudin, Nicholas LoRocco, Xiaoxi Zhuang

**Affiliations:** 1 The Committee on Genetics, Genomics and Systems Biology, The University of Chicago, Chicago, IL 60637 USA; 2 The Committee on Neurobiology, The University of Chicago, Chicago, IL 60637 USA; 3 The Interdisciplinary Scientist Training Program, The University of Chicago, Chicago, IL 60637 USA; 4 The Department of Neurobiology, The University of Chicago, Chicago, IL 60637 USA; 5 The Neuroscience Institute, The University of Chicago, Chicago, IL 60637 USA

**Keywords:** Psychiatric disorders, Neuroscience

## Abstract

Dopamine modulates corticostriatal plasticity in both the direct and indirect pathways of the cortico-striato-thalamo-cortical (CSTC) loops. These gradual changes in corticostriatal synaptic strengths produce long-lasting changes in behavioral responses. Under normal conditions, these mechanisms enable the selection of the most appropriate responses while inhibiting others. However, under dysregulated dopamine conditions, including a lack of dopamine release or dopamine signaling, these mechanisms could lead to the selection of maladaptive responses and/or the inhibition of appropriate responses in an experience-dependent and task-specific manner. In this review, we propose that preventing or reversing such maladaptive synaptic strengths and erasing such aberrant “memories” could be a disease-modifying therapeutic strategy for many neurological and psychiatric disorders. We review evidence from Parkinson’s disease, drug-induced parkinsonism, L-DOPA-induced dyskinesia, obsessive-compulsive disorder, substance use disorders, and depression as well as research findings on animal disease models. Altogether, these studies allude to an emerging theme in translational neuroscience and promising new directions for therapy development. Specifically, we propose that combining pharmacotherapy with behavioral therapy or with deep brain stimulation (DBS) could potentially cause desired changes in specific neural circuits. If successful, one important advantage of correcting aberrant synaptic plasticity is long-lasting therapeutic effects even after treatment has ended. We will also discuss the potential molecular targets for these therapeutic approaches, including the cAMP pathway, proteins involved in synaptic plasticity as well as pathways involved in new protein synthesis. We place special emphasis on RNA binding proteins and epitranscriptomic mechanisms, as they represent a new frontier with the distinct advantage of rapidly and simultaneously altering the synthesis of many proteins locally.

The idea of altering memory or synaptic strength as therapy for neurological and psychiatric disorders has been explored previously. For example, reversing aberrant synaptic long-term depression (LTD) has been proposed as a therapy for Fragile X syndrome [1–3]. Altering fear memory during reconsolidation in post-traumatic stress disorder (PTSD) patients as a therapy represents another example [4, 5]. However, clinical trials based on these ideas have not been successful. Here we argue that circuit-specific correction of aberrant synaptic strength is essential for therapies based on such ideas. This review will focus on corticostriatal synaptic plasticity in the cortico-striato-thalamo-cortical (CSTC) loops. The reason for such a focus is that CSTC loops offer many good examples of how changes in synaptic strength can lead to both pathology and possible therapeutic solutions. These include clinical evidence of therapeutic benefits due to circuit-specific correction of aberrant synaptic strength, animal models of diseases with circuit-specific aberrant synaptic strength, and animal models of circuit specific correction of aberrant synaptic strength, although the existing literature lacks a systematic review devoted to such a focus. This review aims to fill such a gap. We will first briefly review CSTC loops, corticostriatal synaptic plasticity, and signaling mechanisms. Then we will review circuit-specific mechanisms and therapies relevant to various diseases.

## Corticostriatal synaptic plasticity in the cortico-striato-thalamo-cortical loops and signaling mechanisms

According to the classic model of basal ganglia function [6–9], activity of D1 receptor-expressing striatal neurons in the direct ‘Go’ pathway (D1 neurons) increases excitation of cortical activity and facilitates movement. By contrast, activity of D2 receptor-expressing striatal neurons in the indirect ‘No-Go’ pathway (D2 neurons) inhibits cortical activity and movement [9–11]. However, recent studies suggest a more nuanced view of D2 neuron function; in vivo recording studies suggest that D2 neuron activity is also involved in initiating movement, discriminating between motor sequences, and altering motor sequences [12–16]. Nevertheless, direct manipulations of D2 neuron activity by optogenetics or chemogenetics clearly demonstrate their role in motor inhibition [9, 17]. Therefore, even though the circuit level function of the D2 neurons is to inhibit cortical neurons, D2 neuron activity is likely to play important roles in many motor acts and the expression of learned motor skills, as inhibition of specific cortical neurons through the D2 CSTC loops could always be important, even in movement initiation. On the other hand, specific alterations in striatal neuron activity or corticostriatal plasticity predominantly in the D1 or D2 pathway could lead to very distinct symptoms under certain pathological conditions, revealing the opposing yet cooperative roles of D1 and D2 loops, which will be discussed below.

At the cellular level, activation of dopamine receptors on striatal neurons modulates gating of ion channels and, therefore, acutely alters the intrinsic excitability of these neurons [18–20]. It is commonly understood in the field that activation of D1 receptors increases D1 neuron firing, whereas activation of D2 receptors decreases D2 neuron firing [19–21]. However, the literature on this topic, especially in vivo studies, is still limited. Moreover, there are also reports showing that striatal neurons form local synaptic connections through their local axon collaterals, thereby providing strong lateral inhibition on surrounding circuitry [22–24], suggesting a more complex picture of D1-D2 interactions at multiple levels.

Another important function of dopamine is to modulate corticostriatal plasticity in both the direct and indirect pathways [25–29]. Such a mechanism is able to produce cumulative and long-lasting changes in corticostriatal synapses which ensures persistent effects on behavior [30–32]. The role of dopamine in modulating corticostriatal plasticity fits well with the role of dopamine as the prediction error signal in reinforcement learning. Phasic increases and decreases of dopamine release relative to baseline are thought to encode positive and negative prediction error signals respectively [25, 33–36]. These “teaching signals” promote changes in corticostriatal synaptic strength, correcting errors in future responses. Therefore, the timing of regulated dopamine release accompanying activity at the relevant corticostriatal synapses is essential for causing changes in specific synapses to reinforce only the most relevant motor acts in a specific task while inhibiting the others.

It is often hypothesized that the D1 receptor is more sensitive to phasic increase in dopamine release (positive reward prediction error) but is not sensitive to phasic decrease in dopamine release (negative reward prediction error) [33, 37, 38]. This is presumably because the D1 receptor has low affinity for dopamine and is not activated at the baseline condition [33]. In contrast, the D2 receptor is more sensitive to phasic decrease in dopamine release (negative reward prediction error) but is not sensitive to phasic increase in dopamine release (positive reward prediction error) because the D2 receptor has high affinity for dopamine and is already saturated at the baseline dopamine level [33]. However, this hypothesis has been challenged for the lack of evidence on D1 versus D2 receptor affinity for dopamine under in vivo conditions [39–42]. We present below an alternative hypothesis: the effects of phasic dopamine signaling (prediction errors) on learning need to be consolidated, which requires new protein synthesis stimulated by high cAMP levels in D1 and D2 neurons.

Intracellularly, both the dopamine D1 and D2 receptors are strongly coupled to the cAMP pathway [43, 44]. Dopamine mainly stimulates cAMP production in D1 neurons and inhibits cAMP production in D2 neurons. Therefore, cAMP is elevated in D1 neurons during phasic increase in dopamine release (positive reward prediction error). In contrast, cAMP is elevated in D2 neurons during phasic decrease in dopamine release (negative reward prediction error). Thus, we hypothesize that reward prediction error signals elevate cAMP level in D1 neurons, promote LTP and new protein synthesis there and consolidate specific motor memories after learning associated with positive reward prediction error signals. Meanwhile, negative reward prediction error signals elevate cAMP level in D2 neurons, promote LTP and new protein synthesis there and consolidate specific motor memories after learning associated with negative reward prediction error signals.

The striatum is unique in the expression of the calcium/calmodulin (CaCaM)-independent adenylyl cyclase type 5 (AC5) [45–47]. This is distinct from other brain regions such as the hippocampus and cortex that predominantly express the CaCaM-activated cyclase, AC1 [48–50]. There is little or no AC1 expression in the adult striatum [45–47]. Therefore, cAMP production in adult striatum can be highly modulated by G-protein coupled receptors, relying less on CaCaM. This may explain why dopamine signaling plays such a dominant role in the induction and directionality of corticostriatal plasticity [27–29, 51]. Studies, including ours, suggest that the direction and magnitude of plasticity in D1 and D2 neurons are regulated by both the afferent activity and intracellular cAMP [28, 51–53]. For example, high concentrations of dopamine reduce cAMP via D2 receptor activation and facilitate LTD in the indirect pathway [51–53]. Conversely, low dopamine levels increase intracellular cAMP, favoring LTP in the indirect pathway [28, 51]. Based on the above hypothesis, LTP is likely more important in memory consolidation in the striatum, whereas LTD is more likely to play a role in short term memory or indirectly affects memory consolidation through its interactions with LTP.

Taken together, with the above mechanisms, dopamine activation of the D1 receptor during a specific motor response will favor corticostriatal LTP in the direct pathway and therefore future selection of such a response under the same context. In contrast, lack of dopamine activation of the D2 receptor during a specific motor response will favor corticostriatal LTP in the indirect pathway and therefore future inhibition of such a response under the same context. While these mechanisms are important for normal response selection and inhibition, under certain pathological conditions, the same mechanisms can become maladaptive; for instance, the almost complete lack of dopamine in Parkinson’s disease (PD), or unregulated dopamine release in L-DOPA-induced dyskinesia. In this review, we hypothesize that aberrant corticostriatal LTP in the D1 pathway is a key contributor to L-DOPA-induced dyskinesia, obsessive-compulsive disorder, and substance use disorders. In contrast, aberrant corticostriatal LTP in the D2 pathway is at least partially responsible for PD motor symptoms, drug-induced parkinsonism, and depression. Preventing or reversing aberrant corticostriatal LTP in the respective pathways could be therapeutic. We propose that combining pharmacotherapy with behavioral therapy or with deep brain stimulation (DBS) could potentially cause desired changes in selected circuits and synapses, and reverse aberrant corticostriatal plasticity. If successful, one significant advantage of correcting aberrant synaptic plasticity is that the therapeutic effects could be long lasting even after cessation of treatment. Potential molecular targets for the pharmacotherapy component of such therapeutic approaches include the cAMP pathway, synaptic proteins involved in synaptic plasticity, and pathways involved in new protein synthesis.

It is important to point out that the above discussions are limited to the role of phasic increase (reward prediction error) or decrease (negative prediction error) in dopamine release during learning. The role of phasic changes in dopamine is only meaningful if we also understand the role of tonic dopamine. Moreover, phasic decrease in dopamine signaling is certainly dependent on tonic dopamine signaling that precedes it. It is likely that phasic and tonic dopamine release are regulated differently and play distinct roles. At baseline condition, dopamine neurons fire spontaneously and asynchronously at low frequency [54, 55]. Because of the potent GABAergic inhibition, not all dopamine neurons fire spontaneously in the basal condition [56]. Additionally, not all action potentials lead to dopamine release. Only a small percentage of synaptic vesicles belong to the readily releasable pool which is only slowly replenished [57, 58]. Therefore, the tonic extracellular dopamine level is relatively stable and low. In contrast, dopamine neuron burst firing can generate phasic, short and fast dopamine transients. Moreover, burst firing is often synchronized across dopamine neurons which can overwhelm dopamine transporter’s reuptake activity, cause a strong phasic dopamine release, and potentially recruit additional distant receptors [42, 54, 55, 59, 60]. While tonic and phasic signals are generally considered to be distinct dopamine signaling mechanisms, the exact nature of tonic dopamine and its function are still not well defined in the literature. In addition to the above view of tonic dopamine caused by baseline spontaneous asynchronous low frequency dopamine neuron firing, some researchers view tonic dopamine as accumulation of phasic dopamine, therefore reflecting net rate of rewards [56]. In this view, tonic dopamine serves the important function of determining the optimal rate of responding of the animal in a particular environment, implying a tight coupling between motivational states and tonic dopamine [61]. Others also emphasize the potential negative impact of tonic dopamine on phasic dopamine. Tonic dopamine may blunt phasic dopamine signals due to either reduced contrast from the elevated baseline dopamine level or a reduction in receptor sensitivity [62, 63].

Related to the above topic, dopamine release may not always reflect dopamine neuron firing. One important mechanism is that striatal cholinergic interneurons can directly cause dopamine release independent of dopamine neuron firing [64]. These cholinergic interneurons, often referred to as tonically active neurons in primate striatum, fire spontaneously, and this spontaneous firing has been linked to acetylcholine release [65–68]. Furthermore, cortical and thalamic glutamatergic inputs help synchronize the firing of these cholinergic interneurons while dopamine inhibits acetylcholine transients, indicating that glutamate and dopamine serve as distinct, yet complementary, regulatory forces shaping cholinergic interneuron function and, in turn, physiological responses in the striatum [69–71]. However, under in vivo conditions, studies also suggest that dopamine dynamics and reward encoding may not depend on acetylcholine release [70, 71]. Extracellular dopamine and acetylcholine levels fluctuate and do not arise from direct local interactions between them within the striatum [70, 71]. These findings underscore the complexity of striatal signaling and highlight the need for additional research to reconcile these diverse observations.

Striatal cholinergic interneurons have an important impact on both corticostriatal LTD and LTP. The D2 receptor dependence of LTD induction in both D1 and D2 neurons seems due to D2 receptors on cholinergic interneurons [72] while deletion of D2 receptors on D2 striatal neurons has more limited impact on LTD induction [73]. In in vivo studies, it was reported that corticostriatal LTP is dependent on the coincidence of phasic dopamine activation and pauses in cholinergic interneurons [74, 75].

It is also conceivable that dopamine signaling could be subcellularly localized. However, our understanding is still very limited in this regard. There are only a few examples and suggestive evidence. Phosphodiesterase 10 A (PDE10A), the major cAMP PDE in mouse striatum, is localized at the plasma membrane and in dendritic spines close to postsynaptic densities and is associated with the A kinase anchoring protein (AKAP150), PKA, NR2A, NR2B, and PSD95. Affinity of PDE10A to the signaling complexes formed around AKAP150 could be reduced by PDE10A phosphorylation [76]. The regional distribution of DARPP-32 in the rat brain follows the general pattern of dopaminergic innervation; it appears to be concentrated in D1 neurons where it is localized in cell bodies, dendrites, axons, and nerve terminals [77]. Live imaging and computational models suggest maximal effects on cAMP production in secondary dendrites, due to segmental decrease of dendrite diameter. Thus, signaling from dendrites to nucleus is not inversely proportional to the distance [78]. With the development of many new tools, we expect to see much better understanding of subcellularly localized dopamine signaling in the near future.

All the above discussions also suggest that the traditional emphasis on dopamine signaling through volume transmission needs to be revised. Due to technical limitations, it was not possible to accurately measure extracellular dopamine levels close to the synapse or capture its dynamics in behaving animals. However, recently developed genetically-encoded dopamine sensors have dramatically improved the spatiotemporal resolution in measuring extracellular dopamine [79–83].

## Aberrant corticostriatal synaptic plasticity in neurological and psychiatric disorders: loss of control

### L-DOPA-induced dyskinesia (LID)

We will start with LID, the primary detrimental side-effect of PD therapy [84]. Although it has been extensively studied, the underlying mechanism is not well understood [85]. In our view, it is one of the best examples that can be explained by aberrant synaptic plasticity in the CSTC loops. LID refers to the abnormal involuntary movements produced by chronic dopamine replacement therapies in advanced stage PD patients [84]. In early-stage PD, L-DOPA is very effective in controlling PD motor symptoms. This is likely because the remaining dopamine terminals are still able to support regulated dopamine release with precise timing, and L-DOPA, as a dopamine precursor, helps to compensate for the loss of dopamine neurons and terminals [86–89]. In advanced stage PD, however, partially restored dopamine release due to L-DOPA therapy is very different from regulated dopamine release. Due to the significant loss of striatal dopamine terminals and the conversion of L-DOPA to dopamine by non-dopamine neurons, dopamine release is no longer physiological, lacking input dependent and properly regulated firing patterns [87, 90]. Moreover, it has been well documented in animal models of PD that the brain’s capacity for storage and clearance of dopamine is greatly impaired [89]. Therefore, in advanced stage PD, administration of L-DOPA will result in dopamine production and release, but it is no longer regulated with precise timing.

**Fig. 1:**
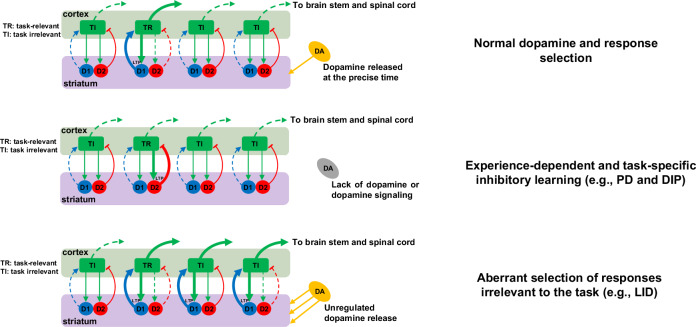
Maladaptive corticostriatal synaptic plasticity in the cortico-striato-thalamo-cortical (CSTC) loops and response selection in diseases. Dopamine modulates corticostriatal plasticity in both the direct “Go” (green) and indirect “NoGo” (red) pathways. Such modulation is usually limited to active corticostriatal synapses (task-specific). Gradual changes in corticostriatal synaptic strengths produce cumulative and long-lasting changes in response selection. Normal phasic dopamine release with precise timing causes changes in corticostriatal synaptic strengths in both “Go” and “NoGo” pathways so that the CSTC loops can facilitate the selection of the most appropriate responses (LTP in the “Go” pathway in task-relevant synapses) and inhibit the rest. Under no dopamine condition (e.g., in PD), excessive corticostriatal LTP in the “NoGo” pathway will lead to experience-dependent and task-specific inhibition. In contrast, unregulated dopamine release (e.g., in advanced stage PD upon L-DOPA treatment) will cause changes in synapses that reinforce motor acts irrelevant to the specific task. Chronically, such aberrant synaptic plasticity could lead to many unwanted motor acts (e.g., dyskinesia).

How will the effects of regulated and unregulated dopamine release on motor control differ? Striatum dependent learning is correlated with changes in corticostriatal synaptic strength [25, 91–93]. Phasic dopamine release is regarded as the prediction error signal, and therefore the teaching signal that causes changes in corticostriatal synaptic strength. Consequently, the CSTC loops can make predictions better in the future and facilitate the selection of the most appropriate responses [33] (Fig. 1). Therefore, the timing of regulated dopamine release is essential for causing changes in specific synapses to reinforce the relevant motor acts in a specific task while inhibiting the others [33–36]. In contrast, due to conversion of L-DOPA to dopamine by non-dopamine neurons in advanced stage PD upon L-DOPA treatment, unregulated dopamine release will likely cause changes in synapses that reinforce motor acts irrelevant to the specific task in our opinion (Fig. 1). Chronically, such aberrant synaptic plasticity could lead to many unwanted motor acts (dyskinesia). Indeed, studies on animal models that examine cellular mechanisms of LID have found aberrant corticostriatal plasticity [94]. In one example, LTP could be de-potentiated in non-dyskinetic rats but not in dyskinetic rats, and the D1 pathway was implicated [95]. In another example, a sub-population of D1 neurons showed abnormally high firing rates evoked by L-DOPA in dyskinetic mice [96]. In a related study from the same group, it was found that a subset of striatal D1 receptor expressing neurons (potentially memory engram cells specific for this type of dyskinesia) were mostly responsible for the dyskinesia as activation of these neurons induced dyskinesia even in the absence of L-DOPA while silencing of these neurons were therapeutic [97].

### Obsessive-compulsive disorder (OCD)

OCD is characterized by intrusive thoughts and repetitive behaviors [98–101]. Functional neuroimaging studies have consistently revealed aberrant activity within the CSTC loops in individuals with OCD [102, 103]. The frontal cortex, striatum, globus pallidus, substantia nigra and thalamus all belong to the CSTC loops that connect discrete parts of the striatum and cortex [6]. These loops are central to both goal-directed actions and habits, which are shaped by striatum dependent learning with changes in corticostriatal synaptic strength as the underlying mechanism [104–108]. The sensorimotor loop, connecting the dorsolateral striatum (DLS) and the sensorimotor cortical areas, plays a more important role in habits and habit learning. In contrast, the more ventral loop, connecting the dorsomedial striatum (DMS) and the association cortices, serves functions related to more flexible goal-directed actions and reward learning. Human behavior and imaging studies have shown an impaired balance between goal-directed behavior and habit learning in OCD patients [109, 110].

Studies on animal models suggest that mutations in synaptic proteins can cause aberrant corticostriatal synaptic plasticity and lead to repetitive, stereotyped, habitual motor behaviors [111, 112]. The otherwise normal role of synaptic proteins in corticostriatal plasticity could become maladaptive with certain mutations and lead to aberrant corticostriatal synaptic plasticity, pathological habitual motor acts and impaired behavioral flexibility. For example, SAP90/PSD95-associated protein 3 (SAPAP3) is a postsynaptic scaffolding protein at excitatory synapses that is highly expressed in the striatum. Mice with genetic deletion of Sapap3 exhibit increased compulsive grooming behavior which are alleviated by a selective serotonin reuptake inhibitor [111], a first-line OCD treatment. Sapap3-mutant mice display defects in cortico-striatal synapses [112]. In the central striatum, postsynaptic responses to inputs from the secondary motor area (M2) were significantly higher in strength and reliability in mutants compared to wild-types, suggesting that increased M2-striatal inputs may contribute to both striatal hyperactivity and compulsive behaviors [112]. Furthermore, lentiviral-mediated expression of Sapap3 in the striatum rescues the synaptic and behavioral defects [111]. DBS that corrects such aberrant synaptic strength in the CSTC loops has been found to be therapeutic in these animal models [113].

Conversely, experimentally induced aberrant corticostriatal synaptic plasticity has been shown to induce OCD-like behaviors in animal models. For example, repeated optogenetic stimulation of the orbitofrontal cortex (OFC)-ventromedial striatum (VMS) projection was reported to progressively increase grooming that persisted even after stimulation cessation [114]. The progressive increase in grooming was correlated with a progressive increase in evoked firing of postsynaptic VMS cells. Furthermore, both increased grooming and evoked firing were reversed by chronic fluoxetine [114]. These data further support the causation of CSTC circuit dysregulation in OCD. However, these studies do not tell us whether abnormal D1 or D2 pathway activity in any of the specific CSTC loops would predict OCD-like behaviors.

Similar to OCD, studies of Tourette syndrome have also suggested that dysfunction in the basal ganglia circuit and corticostriatal synaptic plasticity may contribute to the disorder [115–118], though there is little direct evidence on synaptic plasticity.

### Substance use disorders

Substance use disorders are increasingly recognized as a synaptic disease with maladaptive appetitive associative learning as one fundamental aspect [25, 119–124]. Instrumental action-outcome learning is important in establishing drug-seeking behavior [125–130]. Pavlovian associative learning is important in making otherwise neutral environmental cues acquire strong incentive values; and drug craving can be induced by conditioned environmental cues [34, 129, 131–133].

Almost all addictive drugs are known to either directly or indirectly increase dopamine signaling. The mesolimbic dopamine system, which originates from the ventral tegmental area (VTA) and projects mainly to the nucleus accumbens, is especially implicated in substance use disorders [119–124, 134]. Repeated drug exposure often leads to long-lasting changes in synaptic strengths in the VTA and the nucleus accumbens, such as LTP [25, 26, 122, 124, 135–139]. These changes are thought to contribute to both the development and the persistence of substance use disorders. Like the dorsal striatum, the nucleus accumbens are largely composed of neurons that express dopamine D1 receptors or D2 receptors [140, 141]. Drug induced increases in synaptic strength in the D1 receptor expressing direct pathway have been found to be correlated with drug seeking behaviors [142–145]. In contrast, the D2 receptor expressing-indirect pathway is often implicated in extinction or aversive learning [14, 146, 147]. Our and other groups’ studies have demonstrated that dopamine signaling through AC5 and the cAMP pathway plays a central role in synaptic plasticity and appetitive learning [43, 45, 51, 124, 148, 149]. While we emphasize D1 and D2 neuron’s distinct roles here, we do not rule out the possibility that both D1 and D2 neuron’s firing patterns can still be well correlated with behaviors in both acquisition and extinction of appetitive and aversive learning since activities of both neurons may be important in the expression of specific behaviors.

## Aberrant corticostriatal synaptic plasticity in neurological and psychiatric disorders: lack of movement or motivation

### Parkinson’s disease (PD)

The contribution of aberrant corticostriatal synaptic plasticity to PD motor symptoms has been highlighted by multiple studies [28, 31, 150]. In 6-OHDA lesioned mice, a model of PD, the induction of corticostriatal LTD in the indirect pathway by a high-frequency stimulation protocol (HFS) was impaired. Correcting this impaired LTD was therapeutic [150]. In addition, in mice lacking D2 receptor activation, HFS that would normally induce LTD instead induced LTP [151]. Therefore, when dopamine levels decrease as PD progresses, it favors corticostriatal LTP induction in the indirect “NoGo” pathway and facilitates motor inhibition. However, it’s not clear in those studies if corticostriatal synaptic plasticity is important to motor learning or motor performance.

Studies in our laboratory have led us to develop a new framework [32, 152–155] that challenges the traditional view, which suggests that dopamine neuron denervation causes an acute imbalance between the direct and indirect pathways, leading to impaired motor performance [7, 8]. We have shown in animal models that the combination of dopamine deficiency and motor experience lead to aberrant corticostriatal LTP in the indirect pathway. Consequently, these animals gradually develop task-specific, experience-dependent motor inhibition and deteriorating motor performance—a ‘use it and lose it’ phenomenon that we call “aberrant inhibitory motor learning” [32, 152–155] (Fig. 1). This framework fits with the cellular level function of dopamine as the reward prediction error signal in modulating corticostriatal plasticity in learning [25–29]: responses that are reinforced by dopamine will be selected more in the future whereas responses that are not reinforced by dopamine will be inhibited in the future [32, 33]. In the pathological condition of Parkinson’s disease, dopamine deficiency or dopamine receptor blockade can cause any motor response to undergo experience-dependent, task-specific deterioration. The task specificity corresponds to the fact that corticostriatal plasticity in specific synapses is dependent on specific cortical glutamatergic inputs at that time.

### Drug-induced parkinsonism (DIP)

DIP is the second most common cause of parkinsonism after idiopathic PD [156–161]. Antipsychotic drugs that block the dopamine D2 receptors are the most important and best characterized cause of DIP [156–162]. It was estimated that DIP prevalence might even approach that of idiopathic PD if the use of antipsychotic drugs in the aging population continues to rise [160]. DIP is characterized mainly by rigidity and bradykinesia, similar to the main motor symptoms of idiopathic PD, while other common idiopathic PD symptoms such as tremor and gait instability as well as many nonmotor symptoms are less prominent in DIP [156–158, 163, 164]. The differences between DIP and idiopathic PD suggest that dopamine D2 receptor blockade in DIP mainly causes rigidity and bradykinesia, while degeneration of dopamine neurons in the midbrain and other places (e.g., the enteric nervous system) in PD lead to motor, as well as nonmotor symptoms.

In DIP, parkinsonism lasts for weeks to months after cessation of antipsychotic treatment [165]. The persistence of these effects even in the absence of dopamine receptor blockade suggests that synaptic plasticity is a key contributing factor [30, 166]. The commonly used animal model for DIP is haloperidol-induced catalepsy. Rats or mice treated with haloperidol initially show mild akinesia and rigidity (i.e., catalepsy). However, repeated administration of haloperidol leads to more severe catalepsy (sensitization) [30, 167–170]. Importantly, haloperidol-induced catalepsy and its sensitization are dopamine D2 receptor dependent; and the sensitization effect is also context dependent [30, 167, 168]. Moreover, adenosine A2A antagonists can significantly protect against the development of catalepsy [169, 170]. A2A antagonists can reduce cAMP levels in D2 neurons and prevent the development of corticostriatal LTP [28], suggesting the role of corticostriatal LTP in the indirect pathway in DIP [30]. Taken together, these observations suggest that D2 receptor blockade will increase cAMP levels in D2 neurons; under conditions of specific cortical glutamatergic inputs (i.e., motor experience), LTP will develop at these excitatory synapses onto D2 neurons in the indirect pathway [32, 152] (Fig. 1). Such aberrant LTP will lead to task-specific inhibition and gradual worsening of motor function. In this model, motor performance deterioration in DIP requires both dopamine deficiency and motor experience.

### Depression

Anhedonia, reduced motivation, and lack of energy are common in depression, and are often linked to a dysfunctional dopamine reward pathway [171, 172]. Human brain imaging studies suggest that depression is associated with impaired brain signals for reward learning and reward sensitivity [173]. One meta-analysis compared healthy controls, current or past major depression or bipolar disorder patients, and used reinforcement learning models to isolate reward sensitivity and learning rate [174]. Results suggest that depression and anhedonia reduced reward sensitivity more than learning rate.

Additionally, studies of patients with known dopamine system dysfunction have revealed high rates of depression diagnoses; nearly 40% of PD patients suffer from depression. This comorbidity is strongly associated with decreased activity in the limbic component of the CSTC loops [175]. In contrast, L-DOPA administration is known to lead to impaired impulse controls, which is associated with increased limbic CSTC circuit activity [176]. Together, this evidence suggests a role for dopamine in bidirectionally mediating these limbic and associative circuits.

Beyond clinical observation, animal models of depression allow for direct examination of synaptic changes within the CSTC loops. Chronic restraint stress in mice decreases the strength of excitatory synapses onto nucleus accumbens D1 neurons [177]. Social defeat stress and anhedonia increase excitatory input frequency onto nucleus accumbens D2 neurons, while simultaneously reducing excitatory transmission onto D1 neurons and diminishing their dendritic complexity [178, 179].

While the above studies highlight the importance of dopamine in depression, it is also important to consider how other neurotransmitters, particularly serotonin, may act on these same CSTC loops. Serotonin is the primary neurotransmitter implicated in depression, as evidenced by the serotonergic nature of most antidepressants. Nevertheless, serotonin can alter dopamine signaling through its effect on the activity of dopamine neurons and striatal neurons. More recent studies suggest the role of serotonin in depression is related to encoding appetitive or aversive outcomes [180]. Using fast-scan cyclic voltammetry recording from the striatum of human patients with PD [181], it was found that transiently increased serotonin was mostly correlated with negative reward prediction errors. Furthermore, trial-to-trial serotonin fluctuations were mostly correlated with protective choices (loss aversion) made by subjects following negative reward prediction errors. If this is true, lower serotonin could result in impaired protective mechanisms following negative outcomes [182]. In mice, it has been reported that serotonin neurons develop a slow ramp-up response to the reward-predicting cue, and ultimately remain responsive to the reward, i.e., they respond to both expected and unexpected rewards [183, 184]. In comparison, dopamine neurons increase their response to the cue but reduce their response to the reward when the animal gradually learns to predict the reward based on the cue, consistent with the reward prediction error hypothesis discussed above. The activities of both types of neurons are modulated by reward values whereas stressors substantially reduce the response strength of both neuron types in the nucleus accumbens [183, 184]. However, studies by another group on mice reported that different subsets of serotonin neurons modulated their responses differently in the dorsal raphe, either to reward or punishment, either phasically or tonically [185]. These studies exemplified the challenges in understanding the exact information encoded by serotonin under different environmental conditions, and whether serotonin dysfunction is the main mechanism for depression [186]. Nevertheless, the recent findings on the role of serotonin in appetitive and aversive learning suggest its either direct role in modulating synaptic plasticity in CSTC loops or its indirect role through affecting dopamine signaling.

## Targeting synaptic plasticity as a therapeutic approach

The above discussion highlights the potential role of maladaptive corticostriatal synaptic plasticity in various psychiatric and neurological disorders, suggesting that preventing or reversing aberrant corticostriatal synaptic strength is indeed a promising therapeutic approach. However, such an approach has not been systematically explored. In the following section, we will discuss some limited examples that include pharmacotherapy, DBS or their combination that target synaptic plasticity in the striatum to treat substance use disorders, PD, OCD, and depression. Table 1 summarizes some of the published studies, although it’s not an exhaustive list.

**Table 1:** Evidence for aberrant corticostriatal synaptic plasticity in diseases from clinical studies and animal models.

Disease		Circuits, neurons and synapses involved. Evidence from disease conditions.	Ref.	Circuits, neurons and synapses involved. Evidence from therapies.	Ref.
Loss of control	L-DOPA-induced dyskinesia (LID)	In L-DOPA-induced dyskinetic rats, no corticostriatal synaptic depotentiation was observed. Furthermore, activation of dopamine D1 receptors prevented depotentiation.	[95]	Activation of a subpopulation of D1 neurons caused dyskinesia in the absence of L-DOPA. Inhibition of these neurons ameliorated LID.	[97]
		Dopamine depletion led to increased firing of D2 neurons during immobility in mice, while D1 neuron firing decreased. Moreover, a subpopulation of D1 neurons exhibited abnormally high firing rates that correlated with LID.	[96]		
	Obsessive-Compulsive Disorder (OCD)	In cognitive tasks, OCD patients were more prone to slips of action, suggesting a deficit in goal-directed control and an excessive reliance on habits.	[110]	*Sapap3* knockout mice exhibited impaired behavioral response inhibition and reduced activity of striatal projection neurons. Optogenetic stimulation of the lateral orbitofrontal cortex and its striatal terminals restored both functions.	[113]
		In *Sapap3* knockout mice exhibiting OCD-like behaviors, corticostriatal postsynaptic potentials were impaired. Viral-mediated restoration of *Sapap3* expression in the striatum rescued both synaptic and behavioral deficits.	[111, 112]	Clinical studies found that the anterior limb of the internal capsule, ventral capsule, ventral striatum, nucleus accumbens, ventral caudate, subthalamic nucleus (STN) and the inferior thalamic peduncle were all effective for DBS treatment of OCD. Side effects were mild, transient and reversible.	[207, 209]
		Repeated orbitofrontal cortex-ventromedial striatum (VMS) stimulation in mice led to increased grooming that persisted after stimulation, and was associated with increased evoked firing of postsynaptic VMS cells. Both were reversed by chronic SSRI treatment.	[114]	DBS is effective in patients with severe OCD who are resistant to conventional pharmacological treatments	[208]
Loss of control	Substance use disorders	Optogenetic self-stimulation of VTA dopamine neurons facilitated cue-induced relapse of drug seeking in mice, associated with potentiation of excitatory afferents onto nucleus accumbens D1 neurons.	[128]	A review of 25 animal and 22 human studies (1974–2021) found that DBS of nucleus accumbens, insula, and STN reduced drug use and seeking in animal models. Human studies mostly targeted the nucleus accumbens and generally showed positive outcomes.	[194]
		Repeated cocaine exposure reduced GABAergic inhibition, increasing VTA dopamine neurons’ susceptibility to long-term potentiation (LTP). The chronic exposure to cocaine disrupted the relationship between synaptic enhancement and behavior.	[135, 136]	5 patients received DBS of the nucleus accumbens for treatment-resistant alcohol addiction. All reported a complete absence of craving for alcohol, with no severe or long-standing side effects.	[196]
		Cocaine potentiated excitatory transmission onto D1 neurons in the nucleus accumbens in mice, in parallel with locomotor sensitization. Depotentiation of cortical inputs to the nucleus accumbens by optogenetic stimulation in vivo restored normal transmission and abolished cocaine-induced locomotor sensitization.	[142]	3 patients received DBS of the nucleus accumbens to treat alcohol dependence. Craving was greatly reduced, and all patients achieved prolonged abstinence.	[197]
		Cocaine induced sensitization and potentiation of excitatory inputs onto nucleus accumbens D1 neurons in mice were normalized by acute nucleus accumbens DBS combined with D1 receptor blockade. This intervention produced a long-lasting abolishment of behavioral sensitization.	[143]	DBS targeting the posterior nucleus accumbens and bed nucleus of the stria terminalis in a patient with severe addiction reduced drug craving and consumption.	[198]
		Repeated morphine exposure potentiated excitatory transmission and increased GluA2-lacking AMPA receptor expression in nucleus accumbens D1 neurons in mice. In vivo optogenetic stimulation of infralimbic cortex-nucleus accumbens shell inputs blocked the reinstatement of morphine-induced conditioned place preference.	[144]	Reversing aberrant enhancements of excitatory synaptic inputs onto striatal D1 neurons is therapeutic in animal models of drug addiction (see left column for details).	[142–145, 190]
		Experimentally induced LTP in D1 neurons in vivo caused a long-lasting increase in alcohol-seeking behavior in rats, while experimentally induced LTD decreased alcohol-seeking behavior.	[145]	Propranolol administration before memory reactivation disrupted the reconsolidation of smoking-related memories in smokers and decreased craving.	[228]
		Repeated cocaine administration upregulated the NAc-cAMP system, contributing to tolerance of cocaine reinforcement. Acute inhibition of this system enhanced drug craving and relapse.	[149]	The NMDA receptor antagonist MK-801 impaired drug-seeking related memory reconsolidation, thereby reducing relapse in animal models.	[229–231]
		Repeated alcohol consumption in mice strengthened glutamatergic transmission onto D1 neurons in the striatum. Chemogenetic excitation of these neurons in vivo promoted alcohol consumption behavior.	[190]		
Lack of movement or motivation	Parkinson’s disease (PD)	In mouse models with dopamine neuron lesions, the lack of corticostriatal LTD in the indirect pathway was observed. Pharmacological restoration of this LTD improved motor control.	[150]	A gradual loss of long-duration response (LDR) was observed after cessation of dopamine replacement therapy in PD patients.	[200]
		Discontinuation of L-DOPA treatment in a PD mouse model did not immediately reduce motor task performance. Instead, performance declined gradually and was dependent on continued task exposure.	[153]	LDR to dopamine replacement therapy in PD patients.	[201]
		In mice with dopamine neuron lesions, motor task performance gradually worsened with continued task exposure. Repeated L-DOPA treatment rescued performance, and this improvement persisted despite treatment withdrawal. Both worsening and rescue were task-specific.	[155]	LDR to dopamine replacement therapy in PD patients was prolonged by A2A antagonists.	[202–204]
	Drug-induced parkinsonism (DIP)	Chronic haloperidol treatment promotes dopamine D2 receptor-dependent corticostriatal LTP in mice.	[166]	A2A antagonism protects against haloperidol-induced catalepsy in rats.	[133, 134]
		Sensitization of haloperidol-induced catalepsy in rats is a context-dependent learning phenomenon	[167]	D2 receptor blockade induced gradual and long-lasting motor performance impairment associated with corticostriatal LTP in mice. A2A antagonism protected against this “inhibitory motor learning”.	[152]
		Haloperidol-induced catalepsy is absent in dopamine D2 receptor knockout mice	[168]	Chronic nicotine treatment mitigated the D2 receptor blockade-induced gradual and long-lasting motor impairment in mice.	[154]
				Adenosine receptor antagonists reduced haloperidol-induced catalepsy in rats.	[169, 170]
	Depression	A meta-analysis of reward-learning studies in patients with major depressive disorder (MDD) and controls found that MDD and anhedonia reduced reward sensitivity more than they affected the learning rate.	[174]	Studies have shown that DBS targeting the subcallosal cingulate gyrus, ventral capsule/ventral striatum, medial forebrain bundle, nucleus accumbens, and the anterior limb of the internal capsule is effective in treating treatment-resistant depression.	[212, 213]
		A comprehensive review of the literature found that depression occurs in approximately 40% of PD patients.	[175]	Chronic stress decreased the strength of hippocampus–accumbens synapses and impaired LTP, antidepressant treatment reversed the stress-induced changes.	[214]
		A review of the literature suggested that depression in PD patients seemed associated with decreased limbic CSTC circuit activity, whereas impulse control disorders seemed associated with increased limbic CSTC circuit activity due to dopamine replacement therapy.	[176]		
		Chronic stress decreased the excitatory synapses strength on D1 neurons in the nucleus accumbens in mice.	[177]		
		Social defeat stress and anhedonia increased excitatory transmission onto D2 neurons, while reducing it on D1 neurons and decreasing their dendritic complexity in the nucleus accumbens.	[178, 179]		
		Depressed patients showed a greater influence of aversive Pavlovian cues on both approach and withdrawal actions over instrumental behavior, suggesting disrupted neural circuits that modulate emotional and motivational processing.	[180]		
		In mice, serotonin neurons respond to both reward-predicting cues and rewards, whereas dopamine neurons shift from reward to cue responses during learning. Both neuron types are modulated by reward value and suppressed by stressors in the nucleus accumbens.	[183, 184]		

### Substance use disorders

Correcting aberrant synaptic strength (e.g., by DBS) has been proposed as novel treatments for substance use disorders. In animal models, optogenetic approaches have shown therapeutic potential by restoring circuit function through the reversal of specific forms of synaptic plasticity [187–189]. In one example, cocaine potentiates excitatory transmission in D1-receptor-expressing striatal neurons in mice via ERK signaling, with a time course paralleling locomotor sensitization. Depotentiation of cortical-nucleus accumbens inputs by optogenetic stimulation in vivo restored normal transmission and abolished cocaine-induced locomotor sensitization [142]. Moreover, low-frequency DBS of the nucleus accumbens combined with dopamine D1 receptor blockade abolished cocaine induced behavioral sensitization [143].

Similarly, repeated alcohol consumption in mice resulted in LTP in the D1-receptor expressing striatal neurons [190], and chemogenetic excitation of these neurons in vivo promoted alcohol consumption behavior. In another example, LTP was induced by pairing presynaptic glutamatergic stimulation with optogenetic postsynaptic depolarization in the dorsomedial striatum. Such experimentally induced LTP in D1 striatal neurons in vivo caused a long-lasting increase in alcohol-seeking behavior, while experimentally induced LTD decreased alcohol-seeking behavior [145]. Repeated morphine administration potentiated excitatory transmission in D1 striatal neurons. In vivo optogenetic stimulation of infralimbic cortex-accumbens shell inputs reversed such pathophysiology and blocked reinstatement of morphine-evoked conditioned place preference [144].

Currently, DBS applications for substance use disorder treatment in humans are still limited, but cases have been reported in which DBS alleviated cocaine, methamphetamine, opioids, alcohol, and tobacco abuse [191–194]. For example, DBS in the nucleus accumbens on patients with alcohol addiction significantly decreased the alcohol craving and consumption [195–197]. In another 30-month longitudinal study, nucleus accumbens DBS in a patient with cocaine addiction was able to reduce the drug craving and achieve long-term abstinence [198]. While these cases highlighted the potential of DBS in treating substance use disorders, double-blinded studies are needed to assess treatment efficacy without bias.

### Parkinson’s disease (PD)

Dopamine replacement therapy usually causes an immediate improvement in motor function, known as the short-duration response (SDR), followed by a long-duration response (LDR) that continues to improve motor function and develops slowly over days to weeks [199]. The phenomenon of LDR supports our hypothesis that PD motor symptoms are at least partially due to aberrant corticostriatal synaptic strength, and that preventing or correcting such aberrant corticostriatal synaptic strength could be therapeutic. Our animal data and model suggest that dopamine-dependent corticostriatal plasticity in the indirect pathway, and retention of such corrected synaptic strength for some time in the absence of dopamine, are the mechanisms underlying LDR [32, 152–155]. Conversely, experience-dependent aberrant plasticity explains the gradual loss of the LDR after continued cessation of therapy [32, 152–155].

In one clinical study, PD patients learned a speed-accuracy task in the “on” state (during L-DOPA treatment) and then were tested in the “off” state. Their performance progressively worsened when subsequently tested during the “off” state [200], suggesting gradual loss of the LDR after cessation of therapy. In another study using existing data from more than 350 PD patients, the hypothesis that a dopamine-dependent motor learning mechanism underlies the LDR was tested [201]. To measure LDR, the performance in finger-tapping before daily L-DOPA doses (in the absence of SDR) was compared among three time points: before the initiation of L-DOPA therapy, 9 weeks and 40 weeks after initiation of therapy. Even though there was no difference between dominant and nondominant hands in SDR, more LDR associated improvement was observed in the dominant compared to nondominant hand, and this effect was dose dependent. These data again support our aberrant corticostriatal plasticity hypothesis for PD motor symptoms.

Non-dopamine replacement therapy that targets signaling molecules for corticostriatal plasticity (e.g., the cAMP pathway and downstream signaling molecules) also supports our aberrant corticostriatal plasticity hypothesis. A2A antagonists have been approved by the FDA to be used in combination with L-DOPA therapy. They can prolong LDR in PD patients but are not effective if used alone [202–204]. This aligns with the known effects of A2A antagonists in preventing aberrant corticostriatal LTP in the indirect pathway induced by dopamine deficiency [28, 51].

LDR in PD therapy suggests the under-appreciated potential of preventing and reversing aberrant corticostriatal synaptic strength as therapy. It remains to be demonstrated if direct manipulation of corticostriatal plasticity through DBS or through the combination of DBS and pharmacotherapy could be used as a novel therapy for PD. Although DBS is already successfully used in treating PD, it is limited to acute therapeutic effects; PD symptoms usually quickly return if DBS stops. What we propose here is to alter corticostriatal synaptic strength instead. If successful, such therapeutic effects should be long lasting.

### Obsessive compulsive disorder (OCD) and depression

Selective serotonin reuptake inhibitors (SSRIs) have demonstrated efficacy in reducing OCD symptoms which is often attributed to modulating serotonin levels and normalizing neurotransmission within the CSTC circuitry [205, 206]. Cognitive-behavioral therapy is another cornerstone of OCD treatment in which exposure and response prevention techniques are used to gradually expose individuals to feared situations or thoughts while preventing the accompanying compulsive behaviors [205, 206].

However, approximately 10% of patients do not respond to either of those treatments. With treatment-resistant patients, DBS has shown promise. The most common targets include the ventral capsule/ventral striatum (VC/VS), the anterior limb of the internal capsule (ALIC), and the subthalamic nucleus (STN) [207]. Stimulation of these regions within the CSTC circuitry aims to normalize neural activity, thus alleviating OCD symptoms. Meta-analysis of therapeutic effects of DBS among OCD patients found that such treatments significantly reduced OCD symptoms [208–210].

DBS has also been tested in animal models. As discussed above, deletion of Sapap3 in mice results in excessive grooming and a selective deficit in behavioral response inhibition. Optogenetic stimulation of the lateral orbitofrontal cortex and its terminals in the striatum normalized striatal neuron activity and restored the behavioral response inhibition [113].

Similar to OCD, major depression is most commonly treated with SSRIs. There is limited literature on DBS treatment of major depression, but it has shown its promise [211]. DBS significantly alleviates depressive symptoms in treatment-resistant depression (TRD) patients by targeting the subcallosal cingulate gyrus (SCG), VC/VS, medial forebrain bundle (MFB), and nucleus accumbens core [212]. One study reviewed both preclinical and clinical findings [213]. In preclinical studies, stimulation parameters and neuroanatomical locations could influence DBS-related therapeutic effects, suggesting that the modulatory effects of monoamine neurotransmitters could be the reason for such therapeutic effects. In clinical studies, DBS in the SCG, ALIC and MFB yielded relatively consistent antidepressant response rates [213]. Interestingly, acute responses to DBS often do not predict the long-term therapeutic effects, suggesting that synaptic plasticity may play an important role in the long-term but not the acute therapeutic effects [211]. In animal model studies, chronic stress decreases the strength of hippocampus–accumbens synapses and impairs LTP whereas antidepressant treatment can reverse such aberrant changes [214].

## Erasing “bad memories” during reconsolidation as a therapeutic approach

The above examples are limited to diseases that are at least partially attributable to abnormal basal ganglia functions; and we discussed some examples in which preventing or reversing aberrant corticostriatal synaptic strength represents promising therapeutic approaches.

A related approach is erasing “bad memories” during reconsolidation even though many of the studies are outside of the basal ganglia field. Under the reconsolidation hypothesis, when a memory is recalled, it becomes labile and needs to be reconsolidated which provides a window of opportunity for memory alteration [5, 215–217]. One often discussed example of such an idea is PTSD although clinical trials have not been successful [218, 219]. PTSD is characterized by the re-experiencing of a traumatic event through intrusive memories [98]. The reconsolidation of traumatic memories involves the strengthening of specific synaptic connections in the brain, and disrupting these connections during the process of memory reconsolidation can potentially weaken the traumatic memory and reduce symptoms [5, 215, 216]. Pharmacological interventions and behavioral techniques have been used, including the beta-adrenergic antagonist propranolol. Propranolol, administered immediately after a traumatic event or before memory retrieval, can reduce the incidence of PTSD symptoms [4, 220, 221]. In addition, many preclinical studies have conducted and have provided proof of principle for such approaches [222–225], and for mechanisms of synaptic plasticity involved in extinction learning and exposure therapy which gradually weaken the memory and PTSD symptoms [226].

Targeting reconsolidation could be a promising approach for correcting maladaptive memories in basal ganglia-related disorders as well [227]. In human studies, interfering with the reconsolidation of nicotine-associated memories using propranolol decreased craving for smoking [228]. In preclinical studies, NMDA receptor antagonist MK-801 was able to impair drug-seeking related memory reconsolidation and therefore reduce relapse to drug-seeking behaviors in animal models [229–231].

## Molecular targets to consider

### Targeting signaling pathways involved in synaptic plasticity

If targeting specific aberrant synaptic plasticity is a potential therapeutic approach for neurological and psychiatric disorders, then it is possible to take advantage of the known molecular mechanisms involved in synaptic plasticity. Not surprisingly, in the above discussion, whether to induce corticostriatal LTP or LTD as a therapy, or to prevent memory reconsolidation, drugs associated with specific molecular targets are usually used, alone or in combination with behavioral therapy or with DBS.

There is a vast literature on neurotransmitter receptors, intracellular signaling molecules, synaptic proteins, cytoskeletal proteins, and cell adhesion molecules involved in synaptic plasticity and dendritic remodeling, e.g., NMDA, AMPA, mGluR1/5, CaMKII, AC1/8, AC5, PKA, deltaFosB, ARC, Homer, BDNF, CREB, PSD-95, Shank, Neuroligins, DISC1 and Dynamin etc. There are already many comprehensive review papers on them [217, 232–242]. Both NMDA and mGluR1/5 have been tested as therapeutic targets as discussed in the above examples.

One potential disadvantage of targeting these molecules alone is that they are less likely to be selective to the underlying pathological mechanisms as they are also crucial for normal synaptic mechanisms involved in learning, memory, and other functions. This may partially explain why clinical trials based on these ideas have not been successful [243–247]. DBS alone has similar limitations. Even though DBS has been successful in treating PD, the therapeutic effect stops when the treatment stops. We propose that combining behavioral therapy or DBS with molecular targets offers unique advantages by combining task-relevant circuit selectivity with specific molecular targets to achieve more specificity in correcting aberrant synaptic plasticity. Another potential advantage of such therapies is that the therapeutic effects could be long-lasting even after cessation of treatment, as corrected synaptic strengths would take time to become aberrant again.

### Targeting new protein synthesis, epigenetic mechanisms, RNA binding proteins (RBPs) and RNA modifications

Based on the memory consolidation idea discussed above, one unique class of molecular targets are those regulating protein synthesis. It is known that new protein synthesis is required for long-term changes in synaptic strength and for converting short-term memory to long-term memory [248, 249], including plasticity involved in the basal ganglia [250–252]. Therefore, blocking protein synthesis can block reconsolidation of old memories or consolidation of new memories, either could be potentially therapeutic.

Synaptic activity can rapidly change the synthesis rates of both specific proteins and the overall proteome [253, 254]. Protein synthesis inhibitors or genetic manipulations that affect protein synthesis have been shown to cause decay of LTP or LTD, blocking the animal’s ability to remember after learning in both explicit and implicit memory tasks [255–259]. Indeed, therapeutic approaches have been proposed by taking advantage of protein synthesis inhibition and interfering with memory reconsolidation [5, 260, 261].

All protein synthesis starts from gene expression. Gene expression studies have revealed some of the important pathways involved in learning and memory [262, 263]. Epigenetic mechanisms of gene expression regulation can potentially induce long-lasting changes in the brain that may underlie persistent behavioral abnormalities [263]. Many studies have shown that manipulations of histone acetylation are effective treatment in animal models of substance use disorders and depression [264–267].

Although important, the above mechanisms affect gene expression at transcriptional level, which lacks synaptic specificity. One neuron could have thousands of synapses, but plasticity is often synapse specific. Moreover, the distance between synapses and the soma creates a fundamental challenge for the neuron. A substantial amount of work has shown that local protein synthesis in dendrites is often required, among other mechanisms, for long-term synaptic plasticity [254, 255, 268, 269]. Some mRNA transcripts are selectively localized to dendrites, suggesting mechanisms that control their distribution and translation [270–274].

The key factors that regulate translation temporally and spatially are those that control RNA transport, localization, translation, and degradation. RNA binding proteins (RBPs) are one of the major mechanisms for such regulations [254, 255, 268, 269, 275]. One important example is the Fragile X mental retardation protein (FMRP) [1, 276]. FMRP was first identified as a key protein related to Fragile X syndrome. Loss of FMRP leads to the disorder that causes intellectual retardation and Autism [277]. Functionally, FMRP is an RBP that modulates synaptic plasticity by regulating local translation [276]. *Fmr1* mutant mice show an increased mGluR-dependent LTD in the hippocampus [278]. Furthermore, reducing mGluR5 expression or inhibiting mGluR5 activity in animal models of Fragile X alleviates the disease phenotype [2, 3]. However, clinical trials have not been successful [279, 280]. Another interesting example is disrupting up-frameshift protein (UPF2), a nonsense-mediated mRNA decay (NMD) component downstream of protein translation. Neuron-specific disruption of UPF2 in adult mice was shown to impair synaptic plasticity, learning, and memory, as well as cause perseverative behavior [281].

How do RBPs recognize and bind to specific targets? The affinities of RNAs to RBPs are often regulated by RNA modifications [282]. Although there are many, we want to emphasize N6-Methyladenosine (m^6^A) RNA methylation as an example because it is especially abundant and important in this regard [283–290]. Studies of m^6^A so far embody the concept of “epitranscriptome” and epitranscriptomic regulation. Studies in the past a few years indicate that the m^6^A modification of RNAs affects almost every phase of mRNA metabolism and function, including RNA transport, localization, splicing, nuclear export, stability, and translational efficiency [283–290]. m^6^A is highly enriched in mRNAs in the brain, especially in the adult brain, and it is present in over 4500 mRNAs in the mouse brain [291]. Notably, behavioral training has been shown to increase m^6^A levels in the prefrontal cortex of mice [292].

The functional significance of m^6^A RNA methylation is exerted by three groups of proteins: “writers” (methyltransferases) that install, “erasers” (demethylases) that remove, and “readers” that are RBPs that recognize m^6^A and determine the cellular fate of the modified RNA [283, 290, 293–298]. The identification of these key players in the m^6^A pathway has opened the door to functional studies of m^6^A.

METTL14 is one essential subunit of the m^6^A methyltransferase complex [283]. In our published studies, *Mettl14* gene deletion in dopamine receptor expressing neurons severely impaired motor learning [299]. In another study, the other essential subunit of the m^6^A methyltransferase complex, METTL3, has been found to regulate the efficacy of hippocampus-dependent memory consolidation by promoting the translation of immediate early genes during memory formation [300]. The m^6^A demethylase FTO is highly expressed in the brain and may be dynamically regulated [301]. In human genetic studies, mutations in the *FTO* gene are strongly linked to obesity and diabetes; *FTO* polymorphisms are also implicated in attention-deficit/hyperactivity disorder, Alzheimer’s disease, and abnormal brain volumes [301]. Animal studies indicate that the *FTO* knockout mice have an abnormal behavioral and electrophysiological response to cocaine [302].

m^6^A reader proteins are special RBPs with diverse functions. YTH-domain containing reader proteins (YTHDF1, 2 and 3) are found at the dendrites of mouse hippocampal neurons, and loss of these proteins results in dysfunction of synaptic transmission [303]. In *Drosophila*, YTHDF reader proteins are also found to be critical to memory formation [304]. These data suggest that the m^6^A methylated mRNA near the synapse can potentially be recognized and regulated by these reader proteins. Among them, YTHDF1 is especially important to neurons. YTHDF1 plays important roles in promoting synaptic protein synthesis (e.g., CaMKIIα) in response to stimuli, as well as in synaptic plasticity and learning [305]. Interestingly, studies also reveal that FMRP preferentially binds to m^6^A-containing RNA and represses protein synthesis through interactions with YTHDF1 [306–309]. Therefore, if FMRP indirectly inhibits the translation of YTHDF1 target transcripts through this interaction, it may have significantly more target transcripts than previously recognized. This suggests the broader importance of FMRP as a translation repressor in the brain, and that therapies targeting RBPs must be tailored to specific cell types or administered within certain time windows associated with aberrant synaptic plasticity, or both, in order to minimize side effects.

By deleting YTHDF1 selectively in D1 and D2 striatal neurons [295, 296], our own studies have found that YTHDF1 deficiency in D1 neurons selectively impaired the acquisition of motor skill learning whereas YTHDF1 deficiency in D2 neurons virtually eliminated inhibitory motor learning in PD models and haloperidol-induced catalepsy in drug-induced parkinsonism models. Moreover, YTHDF1 deficiency mimics METTL14 deficiency in a cell type specific manner [296], suggesting that YTHDF1 is the main m^6^A reader protein that mediates m^6^A’s function in learning.

Therefore, targeting protein synthesis pathways (e.g., inhibiting YTHDF1) to prevent memory consolidation or reconsolidation could be therapeutic. There are many potential molecular targets to consider. Among them are the well-studied pathways involved in gene expression regulation including epigenetic mechanisms. Here, we argue that RBPs and epitranscriptomic mechanisms represent a new frontier and have the advantage of rapidly altering local protein synthesis in synapses. We expect that this new research direction will yield significant opportunities for the development of novel therapies.

## Conclusion

There are many neurological and psychiatric disorders in which aberrant synaptic plasticity has been implicated. These studies represent an exciting new theme in translational neuroscience and promising new directions for therapy development. However, merely labeling a disease condition as aberrant plasticity is not helpful. We need to understand the specific neural pathways and synapses affected by the pathology; we need to know how specific alterations (strengthening, weakening or other modulations) in synaptic strengths of specific circuits lead to *predictable* behavioral outcomes based on known circuit function. Only then can we harness the power of synaptic plasticity as therapy rather than fall victim to the devastating consequences of aberrant synaptic plasticity.

One potential advantage of correcting aberrant synaptic plasticity is that the therapeutic effects could be long lasting even after cessation of treatment, as corrected synaptic strengths would take time to become aberrant again. It is conceivable that such approaches, if they work as predicted, could have fewer side effects. Even if the acute treatment itself has side effects, they would be short-lived whereas the corrected synaptic strength and the associated therapeutic effects would be relatively long-lasting.

One of the most important advances in modern neuroscience research is the development of optogenetic and chemogenetic tools, which enable precise manipulation of neural circuits and synapses to produce specific behavioral changes [310–312]. While these tools are not yet practical for human therapy, they nevertheless have shown us the promise of direct and selective circuit manipulations compared to DBS, which often lacks cell-type specificity. An intriguing possibility in humans is the combination of pharmacotherapies with direct brain circuit manipulations or specific behaviors, which could potentially be far more selective in correcting aberrant synaptic plasticity than either approach alone. Such approaches fit with the known procedures used in inducing LTP or LTD. They also fit with the idea of combining specific behaviors with drugs that interfere with memory reconsolidation such that the specific memories will become labile and prone to alteration. In this regard, we see a very promising future in combining decades of research on molecular and pharmacological targets with the more recent research on direct circuit manipulations.
